# Disinhibition of Cathepsin C Caused by Cystatin F Deficiency Aggravates the Demyelination in a Cuprizone Model

**DOI:** 10.3389/fnmol.2016.00152

**Published:** 2016-12-21

**Authors:** Junjie Liang, Ning Li, Yanli Zhang, Changyi Hou, Xiaohan Yang, Takahiro Shimizu, Xiaoyu Wang, Kazuhiro Ikenaka, Kai Fan, Jianmei Ma

**Affiliations:** ^1^Graduate School of Dalian Medical University Dalian, China; ^2^Cardiovascular Division, Hailar People's Hospital Hailar, China; ^3^Department of Surgery, Wafangdian Central Hospital Dalian, China; ^4^Department of Anatomy, Dalian Medical University Dalian, China; ^5^Liaoning Provincial Key Laboratory of Brain Diseases Dalian, China; ^6^Richardson Lab, University College London London, UK; ^7^Department of Linguistics and Modern Languages, Chinese University of Hong Kong Shatin, Hong Kong, China; ^8^Department of Neurobiology and Bioinformatics, National Institute for Physiological Sciences Aichi, Japan

**Keywords:** cystatin F, cathepsin C, microglia, CXCl2, demyelination, cuprizone

## Abstract

Although the precise mechanism underlying initial lesion development in multiple sclerosis (MS) remains unclear, CNS inflammation has long been associated with demyelination, and axonal degeneration. The activation of microglia/macrophages, which serve as innate immune cells in the CNS, is the first reaction to even minor pathologic changes in the CNS and is considered an initial pathogenic event in MS. Microglial activation accompanies a variety of gene expressions, including cystatin F (Cys F), which belongs to the cystatin superfamily and is one of the cathepsin inhibitors. In our previous study we showed that Cys F has a unique expression pattern in microglia/macrophages in the demyelination process. Specifically, the timing of Cys F induction correlated with ongoing demyelination, and the sites of Cys F expression overlapped with areas of remyelination. Cys F induction ceased in chronic demyelination when remyelination capacity was lost, suggesting that Cys F expressed by microglia/macrophages may play an important role in demyelination and/or remyelination. The functional role of Cys F in demyelinating disease of the CNS, however, is unclear. Cys F gene knockout mice were used in the current study to clarify the functional role of Cys F in the demyelination process in a cuprizone-induced demyelination animal model. We demonstrated that absence of the Cys F gene and the resulting disinhibition of cathepsin C (Cat C) aggravates the demyelination, and this finding may be related to the increased expression of the glia-derived chemokine, CXCL2, which may attract inflammatory cells to sites of myelin sheath damage. This effect was reversed by knock down of the Cat C gene. The findings gain further insight to function of Cat C in pathophysiology of MS, which may have implications for therapeutics for the prevention of neuroinflammation-involved neurological disorders in the future.

## Introduction

Myelin is a spiral multi-layered structure that is wrapped around the neuron axonal surface. Myelin is produced by oligodendrocytes and is characterized by important effects, such as accelerating nerve conduction velocity, insulation, and neurotrophy in the central nervous system (CNS). Myelin of the CNS is susceptible to a variety of metabolic, toxic, and autoimmune insults, which can lead to demyelination of axons and result in dysfunctional movement, cognition, and sensation. Multiple sclerosis (MS) is a common demyelinating disease of the CNS. Although the precise mechanism underlying initial lesion development in MS is unclear, the CNS inflammation is characterized by increased glial activation, pro-inflammatory cytokine concentrations, blood-brain-barrier permeability, and leukocyte invasion, and long been associated with demyelination and axonal degeneration, which are the hallmarks of MS (Lassmann et al., 2007). The activation of microglia/macrophages, which serves as innate immune cells in the CNS, is the first reaction to even minor pathologic changes in the CNS (Kreutzberg, 1996) and is considered an initial pathogenic event in MS (Singh et al., 2013). Microglial activation accompanies a variety of gene expressions and may cause myelin/axonal damage. Alternatively, microglia may clean up damaged myelin/axonal debris, which promotes remyelination (Lampron et al., 2015). In our previous study, using cDNA microarray analysis and other molecular biology and morphologic methods, we found changes in the expression of numerous genes during the demyelination process, such as cathepsins, which have been shown to play a significant role in inflammatory responses, induction of cytokines, and tissue damage (Ma et al., 2007; Conus and Simon, 2010; Perišić Nanut et al., 2014). The cathepsins family is found primarily in lysosomes and plays key roles in intracellular degradation of proteins and peptides. This view has been broadened because cathepsins are involved in a number of important cellular processes, such as antigen presentation, bone resorption, apoptosis, and protein processing, as well as several pathologic events, such as cancer progression, inflammation, and neurodegeneration (Magister and Kos, 2013). Under physiologic conditions, the activity of the cathepsins is strictly regulated by cystatins, which are the endogenous inhibitors of cathepsins and act as one of the main means of regulation. A broad spectrum of biological roles has been suggested for cystatins, including a role in protein catabolism, regulation of hormone processing and bone resorption, inflammation, antigen presentation, and T-cell dependent immune responses, and resistance to various bacterial and viral infections (Magister and Kos, 2013). Cystatins have been suggested to serve as modulators of the proteolytic system in several diseases (Magister and Kos, 2013), including demyelinating diseases (Ma et al., 2007, 2011; Sladkova et al., 2011; Duan et al., 2012). Cystatin F (Cys F), which belongs to the cystatin superfamily, is a cathepsin inhibitor. In our previous study we reported that Cys F has a unique pattern of expression in microglia/macrophages in the demyelination process. The timing of Cys F induction correlated with ongoing demyelination, and the sites of Cys F expression overlapped with the remyelination areas. Induction of Cys F ceased in chronic demyelination when the remyelination capacity was lost, suggesting that Cys F expressed by microglia/macrophages may play an important role in demyelination and/or remyelination (Ma et al., 2011). Predating our study, Cys F was identified as an up-regulated gene after lipopolysaccharide (LPS) stimulation of monocyte-derived dendritic cells (Hashimoto et al., 2000) and was independently designated as a cystatin-like metastasis-associated protein, the level of expression of which is correlated with metastatic potential in liver tumors (Morita et al., 2000). We have described in detail the pattern of Cys F expression in microglia/macrophages in the CNS of several demyelinating animal models and in the spinal cord tissues of MS patients (Ma et al., 2011); however, the functional role of Cys F in demyelinating diseases of the CNS is unclear.

Using Cys F gene knockout mice, we clarified the functional role of Cys F during the demyelination process in a cuprizone-induced demyelination animal model. We demonstrated that lack of the Cys F gene and the resulting disinhibition of *cathepsin C* (Cat C) aggravates the demyelination status, and this may be related to increased expression of the glia-derived chemokine, CXCL2, which may attract inflammatory cells to sites of myelin sheath damage. This effect was reversed by knock down of the Cat C gene.

## Materials and methods

### Animals

Homozygote B6 and 129-Cst7^tm1Ayr^/J (Cys F KO) mice were purchased from the Jackson Laboratory (stock no. 008157) and CatC STOP-tetO mice (Cat STOP-tetO/+; C57BL/6 background) and Iba1-tTA mouse line 75 (initial BDF1 background backcrossed to C57BL/6 background) were kindly provide by Professor Ikenaka. Cys F KO mice and their wild-type littermates were used after mating with C57BL/6 mice (provided by Dalian Medical University) repeatedly for five generations. Genotyping of the Cys F KO, Iba1-tTA, and CatC STOP-tetO mice were determined by restriction fragment length polymorphism analyses. The primers used for mouse genotyping are shown in Table 1. Cys F KO and Cat C^*STOP*−*tetO*/*STOP*−*tetO*^ (Cat C KD) mice were used to establish a cuprizone-induced demyelination model by feeding 0.2% cuprizone (Sigama, MO, USA) for 4 weeks. The wild-type littermates were used as a control group. All procedures were in accordance with the Dalian Medical University Guidelines for the Care and Use of Laboratory Animals and were approved by the Laboratory Animal Care and Use Committee of Dalian Medical University. Each group had at least three mice. All efforts were made to minimize animal suffering.

**Table 1:** **Sequences of primers for real time-PCR**.

**Gene**	**Sequences of primers**	**Product size (bp)**
*cytatin F*	5′-AGGAAAGGAAGAGGGTTGCCTGAA-3′	415
	5′-TCATGTGTTCATGGTTGGGAGGGA-3′	
*neomysin*	5′-CTTGGGTGGAGAGGCTATTC-3′	280
	5′-AGGTGAGATGACAGGAGATC-3′	
*cathepsin C*	5′-TTCCACGGAGTCAGAAATGCAGGA-3′	1283
	5′-GAGCCAAGTGTTAGGCATTGCGTT-3′	
Cat C STOP-tetO	5′-AGCAGAGCTCGTTTAGTGAACCGT-3′	895
	5′-GAGCCAAGTGTTAGGCATTGCGTT-3′	
Iba1-tTA	5′-ATGCCTGGGAGTTAGCAAGGGAAT-3′	380
	5′-CGGAGTTGATCACCTTGGACTTGT-3′	

### Tissue preparation

Mice were anesthetized with 0.4% chloralhydrate, then some were perfused with cold phosphate-buffered saline (PBS; Coolaker, Beijing, China). The brains were removed for protein and mRNA collection. Other mice were perfused with 4% paraformaldehyde in PBS solution. The brains were removed and stored at 4°C in the same fixative overnight. Then, the brains were transferred into PBS containing 20% sucrose (Amrzsco, OH, USA) overnight, embedded in OCT compound (Sakuro Finetek, CA, USA), and cut into 18 μm slices. Coronary brain sections were cut for IHC and myelin staining.

### Cell cultures

The primary mixed glial cells were prepared from neonatal C57BL/6J mouse pups as reported previously (Ma et al., 2011). In brief, after carefully removing the meninges, the neonatal brain was disintegrated by pipetting. The cell suspension was seeded in 10-cm culture dishes at a density of one brain per dish. Dulbecco's modified eagle medium (DMEM, 10 mL; Sigma, St. Louis, MO, USA) containing 10% fetal bovine serum (FBS; ICN Biomedicals, Aurora, OH, USA) was added to each dish. After14 days *in vitro*, primary mixed glial cells were dissociated by trypsinization, and the cell suspension was plated in 15.6-mm culture dishes for treatment with Cat C (R&D Systems, Minneapolis, MN, USA); each culture dish contained 1 × 10^6^ cells.

### Immunohistochemical (IHC) staining

IHC analysis was performed as described previously (Ma et al., 2011). The following antibodies were used: rat anti-PLP monoclonal antibody (1:1, clone AA3) (Yamamura et al., 1991), goat anti-Cat C antibody (1:100 R & D Systems), rabbit anti-Iba-1 polyclonal antibody (1:500; Wako, Osaka, Japan), rat anti-myelin basic protein monoclonal antibody (1:200; Abcam, Hong Kong, China), and mouse anti-adenomatous polyposis coli (CC-1) monoclonal antibody (1:50; Calbiochem, Darmstadt, Germany). Secondary antibodies were labeled with biotin antibodies (1:200; Vector Laboratories Inc., Burlingame, CA, USA). After the IHC reaction, images were captured using a Nikon digital camera system (DS-Fil) united with microscopy (Nikon eclipse 80i; Tokyo, Japan).

### *In situ* hybridization

The method of *in situ* hybridization (ISH) was performed as described previously (Ma et al., 2006). We used digoxigenin (DIG)-labeled Cat C (NM_009982, 193–1324 bp), *c-fms* (EST clone, AA473814, Invitrogen, CA, USA) cRNA probes. Samples were incubated overnight with alkaline phosphatase-conjugated anti-DIG antibody (Roche, Basel, Switzerland) at 4°C. After hybridization of antisense cRNA probes, dyeing was completed by incubation with 4-nitro blue tetrazolium chloride/5-bromo-4-chloro-3-indolyl-phosphate (NBT/BCIP) (Roche Diagnostic Gmbh, Mannheim, Germany) for 16 h at room temperature. Some sections were then stained with Nuclear Fast Red for survey and analysis. Three sections from each mouse were used for analysis.

### Myelin staining

Black-gold staining was performed according to a protocol adapted from the manufacturer (Millipore, Billerica, MA, USA). In brief, three sections from each mouse were stained in pre-heated 0.3% black-gold solution in a 65°C incubator for 45 min. The sections were then immersed in 1% sodium thiosulfate solution in a 65°C incubator for 20 min.

### DNA extraction and genotyping

The DNA was extracted from the mouse tail using a heat extraction method (Ma et al., 2011). The PCR was performed in 10 μl volumes containing 1 ul dNTPs (2.5 mM), 0.5 μl of each primer, 0.05 μl of Taq DNA polymerase, 1 μl of 10 × buffer with 0.5 μl of DNA, and Milli-Q water. Amplification was performed using reaction components with an initial denaturation at 95°C for 1 min, then 39 cycles at 95°C for 30 s, 60°C for 30 s, and 72°C for 45 s, followed by extension at 72°C for 60 s. The PCR outcome was subjected to 2% agarose gel electrophoresis and the consequences were analyzed by a gel imaging and analysis system.

### Real-time quantitative PCR

Total RNA was extracted from the brain and primary mixed glial cells (1 × 10^6^) with Trizol reagent (Invitrogen, CA, USA) according to the manufacturer's protocol. The RevertAid First Strand cDNA Synthesis Kit (Thermo Scientific Grand Island, NY, USA) was used to synthesize cDNA from 1 μg of total RNA. The cDNA was resuspended in 20 μl of H_2_O, and 2 μl of cDNA samples were used for real-time PCR in a total volume of 25 μl with SYBR Green Reagent (Thermo Scientific Grand Island, NY, USA), ROX (Thermo Scientific Grand Island, NY, USA) and specific primers (Table 1). Results for each sample were normalized to the concentration of β-actin mRNA measured in the same samples and expressed as the fold increase over baseline levels on control samples from naïve mice and untreated mixed glial cells, which were set at a value of 1.

### Enzyme-linked immunosorbent assay (ELISA)

Cys F KO and littermate wild-type mice were treated with cuprizone for 4 weeks. Total brain protein was extracted using a KeyGEN Nuclear and Cytoplasmic Protein Extraction Kit (KeyGEN BioTECH, Nanjing, China) following the manufacturer's protocol. The other samples were primary mixed glial cells under the same conditions as above, prior to treatment with different doses of Cat C (560, 56, 5.6, and 0.56 ng) for 24 h. After treatment, a MIP-2 (CXCL2) ELISA Kit (Abcam) was used for quantitative analysis of MIP-2 in brain and the primary mixed glial cell cultures, according to the manufacturer's protocol.

### Image analysis

After black-gold or PLP and Cat C IHC staining, according to the mouse brain atlas (Keith B. J. Franklin & George Paxinos), three coronary brain sections per animal around the bregma (−1.46 to −1.50 mm) were selected, then three high magnification images were captured randomly in the corpus callosum (CC) using identical exposure times and camera settings to quantify the myelin status and Cat C-positive area using Image J (National Institutes of Health) software. The main process includes changing the color images into 32 bit black-white images, then three rectangular areas per image were randomly selected, the threshold was set, and the staining signal existing area was labeled and measured. The ratio value of the MBP, PLP, or Cat C positive signal area to the total area in the rectangle was used for statistical analysis. Additionally, three or four mice were chosen for calculation of c-fms (*n* = 4) and Cat C (*n* = 3) positive signals in ISH staining, as well as CD 45 (*n* = 4) positive signals in IHC staining. Three high magnification images were captured in the middle part of CC in each mouse, and three fields were chosen randomly in each image. NIS-Elements 3.0 software was used to calculate the number of positive signals per mm^2^ field.

### Statistical analysis

Data are expressed as the mean ± standard error of the mean (SEM) from three independent experiments. All statistical analyses were performed using the Statistical Package for Social Sciences (version 11.5). The data were evaluated for statistical significance with a one-way ANOVA. *P* < 0.05 was considered statistically significant.

## Results

### Cuprizone-induced demyelination was more severe in Cys F KO mice

**Figure 1:**
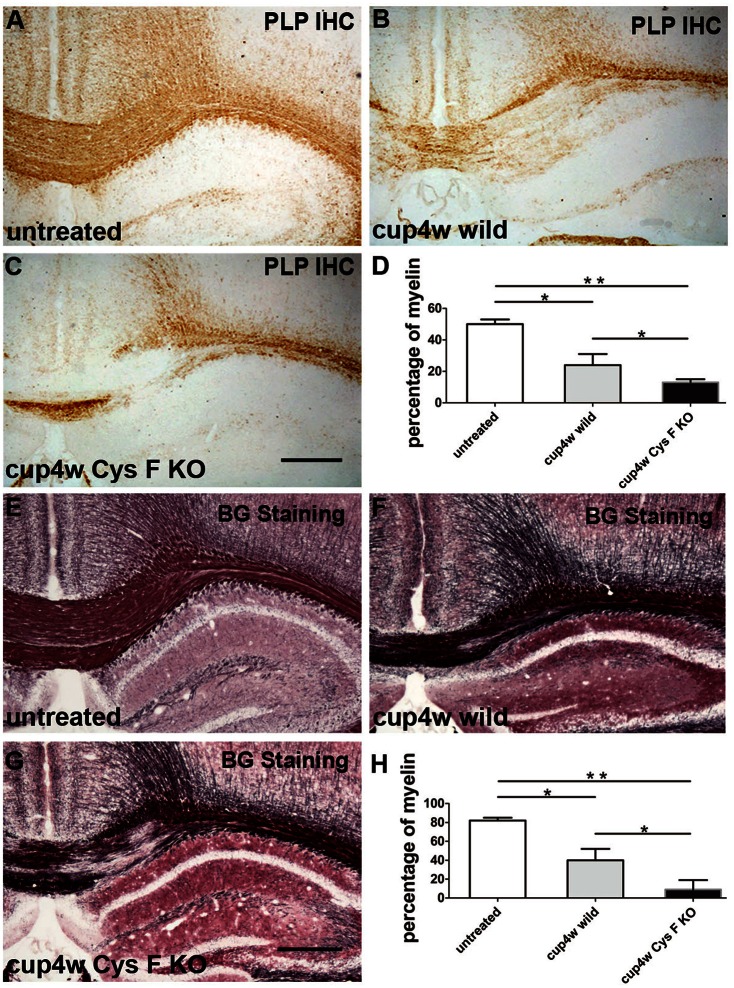
**The remaining myelin status in wild type and Cys F KO mice after cuprizone treatment for 4 weeks**. PLP IHC and Black Gold staining were performed in the untreated mice **(A,E)**, cuprizone-treated wild type mice **(B,F)** and Cys F KO mice **(C,G)**, respectively. Percentage of remaining myelin in corpus callosum is shown in **(D,H)**. ^*^P < 0.05, ^**^P < 0.01. *n* = 6 per group. Scale bar in **(A–C)** and **(E–G)**: 200 μm.

To clarify the functional role of Cys F in the demyelination process, a cuprizone-induced acute demyelinating model was generated in wild and Cys F KO mice. According to the time window in the cuprizone model (Mason et al., 2001), most axons were demyelinated after 4 weeks of a cuprizone diet. Thereafter, remyelination coincided with demyelination even in the presence of continuous cuprizone. To distinguish demyelination from the remyelination process, the demyelination status was analyzed by PLP IHC (Figures 1A–C) and Black Gold staining (Figures 1E–G) in the present study after the mice were fed with cuprizone for 4 weeks; untreated wild-type and Cys F KO littermates were used as the control. There were no significant differences between the untreated wild type and Cys F KO mice with PLP IHC or Black Gold staining (Supplementary Figures 1A–C,G,H,J), while after cuprizone feeding, massive demyelination in the corpus callosum and bits of demyelination in the hippocampus occurred in Cys F KO and wild-type mice. Quantitative comparison of the residual myelin (Figures 1D,H) showed that Cys F KO mice had more robust demyelination than wild type mice following cuprizone feeding. These results indicate that knockout of the Cys F gene aggravates cuprizone-induced demyelination in the brain.

### Cat C expression was up-regulated in Cys F KO mice fed with cuprizone for 4 weeks

**Figure 2:**
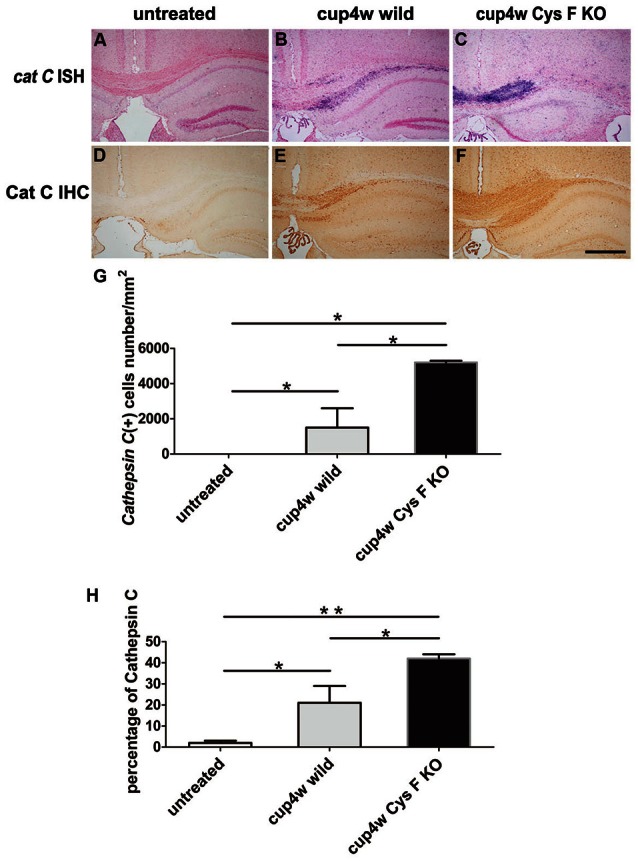
**The expression of Cat C mRNA and protein in corpus callosum in wild type and Cys F KO mice treated by cuprizone for 4 weeks**. Cat C ISH **(A–C)** and Cat C IHC **(D–F)** staining were performed in the untreated mice **(A,D)**, cuprizone-treated wild type **(B,E)** and Cys F KO mice **(C,F)**. Percentages of Cat C ISH and Cat C IHC positive cells in corpus callosum are shown in **(G,H)**, respectively. ^*^P < 0.05, ^**^P < 0.01. *n* = 3 per group. Scale bar in **(A–F)**: 200 μm.

Previous reports have shown that Cat C is a major target of Cys F in different immune cell types. Cat C can activate serine proteases in T cells, natural killer cells, neutrophils, and mast cells (Hamilton et al., 2008). Also, we previously found Cat C is predominantly expressed in hippocampal CA2 neurons in normal mice, but up-regulated in activated microglia in neuroinflammation induced by LPS intraperitoneal injection (Fan et al., 2012). Taken together, we believed that knockout of Cys F aggravated cuprizone-induced demyelination might be related to Cat C expression. Therefore, we first performed *in situ* hybridization (ISH; Figures 2A–C) and IHC staining (Figures 2D–F) for Cat C. No Cat C-positive signals in CC and cortex in untreated mice, including wild type (Figures 2A,D) and Cys F KO mice (Supplementary Figures 2A,B), but massive Cat C expression was seen in demyelinated areas, such as the CC and cortex. Cat C mRNA-positive cells (Figure 2G) and the ratio of the Cat C IHC-positive area-to-total area (Figure 2H) in the CC were quantified for statistical analysis after cuprizone treatment for 4 weeks. Cat C-positive cells in Cys F KO mice were significantly greater than wild type mice (Figures 2G,H). These results confirmed our speculation that severe demyelination in Cys F KO mice might be related to up-regulated Cat C expression in demyelinated areas.

### More microglia/macrophages accumulated in the cuprizone-induced demyelinated areas in Cys F KO mice

**Figure 3:**
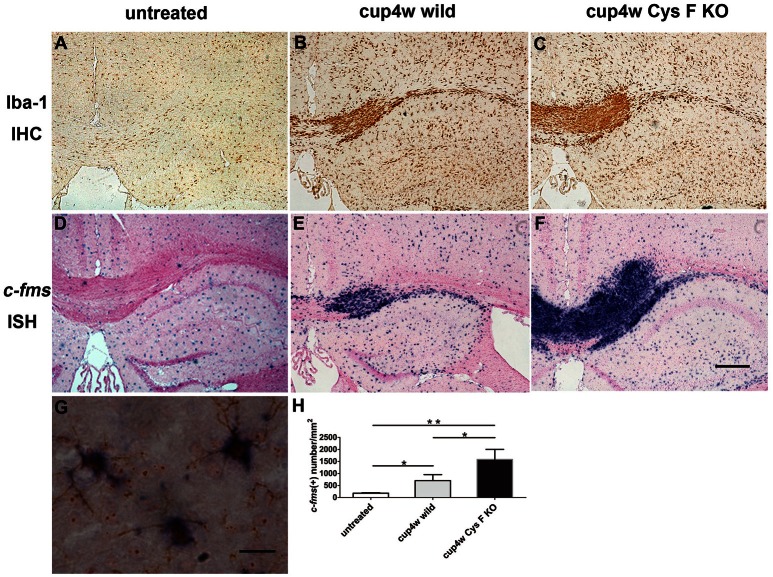
**The accumulation of microglia/macrophagesin corpus callosum in wild type and Cys F KO mice after cuprizone treatment for 4 weeks**. Iba-1 IHC **(A–C)** and *c-fms* ISH **(D–F)** staining were performed in untreated mice **(A,D)**, 4 week-cuprizone treated wild type **(B,E)** and Cys F KO mice **(C,F)**. The double staining of Cat C IHC and Iba-1 IHC staining is seen in **(G)**. Quantitative analysis result of *c-fms* positive cells in corpus callosum is shown in **(H)**. ^*^P < 0.05, ^**^P < 0.01. *n* = 4 per group. Scale bar in **(A–F)**: 200 μm.

In our previous study, the up-regulated expression of Cat C was mainly found in microglia/macrophage lineage cells in LPS–induced neuroinflammation (Fan et al., 2012). To further confirm the cellular source of Cat C, we performed *c-fms* ISH and Iba-1 IHC staining, which are commonly used as a marker of microglia/macrophages, to detect microglia/macrophages in wild and Cys F KO mice after cuprizone treatment. After 4 weeks of cuprizone feeding, masses of microglia/macrophages accumulated at the demyelinated areas (Figures 3A–F), and the number in Cys F KO mice overwhelmed the wild-type littermates (Figure 3H). However, the number of microglia/macrophages in untreated Cys F KO mice were similar to that in the untreated wild-type littermates (Supplementary Figures 3A–F). Further, double staining of Cat C ISH and Iba-1 IHC staining demonstrated that Cat C is expressed in microglia/macrophages (Figure 3G). These data demonstrated that up-regulated expression of Cat C reflects the increased density of microglia/macrophages in Cys F KO mice.

**Figure 4:**
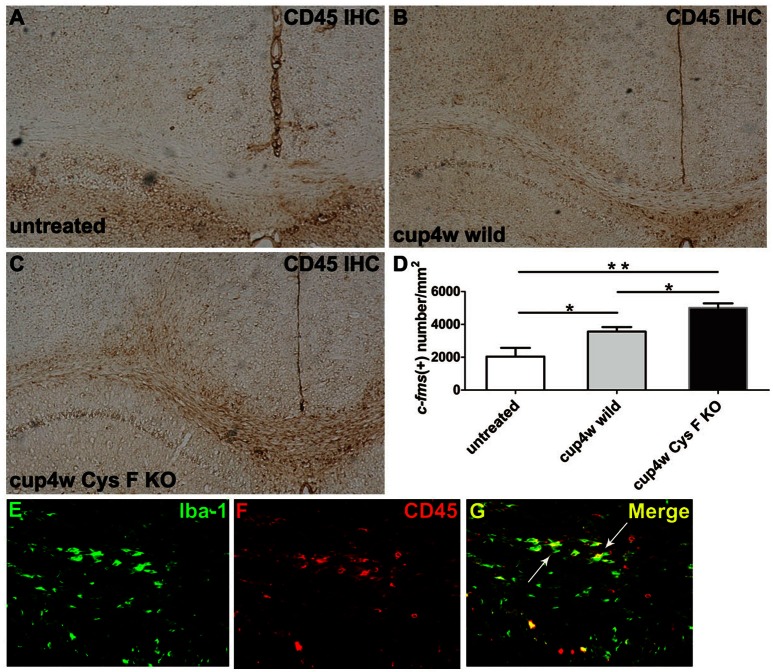
**CD45 positive cells in wild type and Cys F KO mice treated by cuprizone for 4 weeks**. CD45 IHC was performed in untreated wild type mice **(A)**, 4 week- cuprizone treated wild type **(B)** and Cys F KO mice **(C)**, respectively. Quantitative analysis of CD45 positive cells in corpus callosum is shown in **(D)**. Iba-1 and CD45 immunofluorescence staining is shown in **(E–G)**, the arrows showed Iba-1 and CD45 double staining cells. ^*^P < 0.05, ^**^P < 0.01. *n* = 4 per group. Scale bar in **(A–C)**: **(E)** 200 μm; in **(E)** 10 μm.

Additionally, we performed CD45 IHC staining. CD45 is the leukocyte common antigen expressed by all hematopoietic cells, except erythrocytes. We found that in normal CC, there were almost no CD45-positive cells (Figure 4A), while a large number of CD45-positive cells appeared in mice fed cuprizone for 4 weeks (Figures 4B,C). Moreover, the number of CD45-positive cells in Cys F KO mice was much greater than wild-type littermates (Figure 4D). Meanwhile, no significant difference for the number of CD45-positive cells was found between untreated Cys F KO mice and wild-type littermates (Supplementary Figures 4A–C). Furthermore, using Iba-1 and CD45 double staining in Cys F KO mice after cuprizone treatment (Figures 4E–G), we found that almost all of the Iba-1-positive cells were CD45-positive (shown by arrowhead). These results demonstrated that leukocytes including monocytes/macrophages aggregated in the cuprizone-induced demyelinated areas in Cys F KO mice.

### Expression of CXCL2 was clearly increased after cuprizone treatment in Cys F KO mice

**Figure 5:**
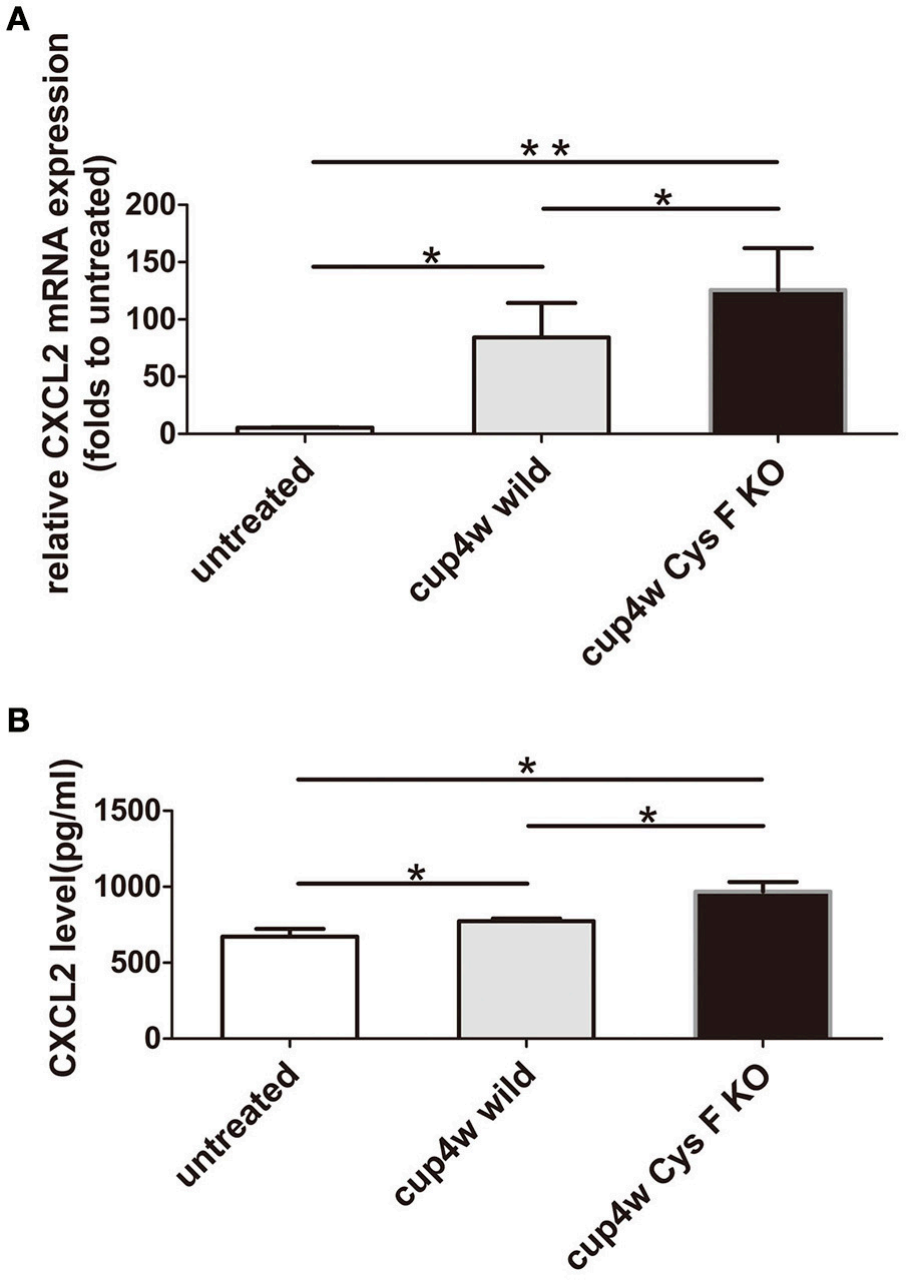
**The mRNA and protein expression of CXCL2 in wild type and Cys F KO mice after 4-week cuprizone treatment**. RNA and protein were isolated from brain tissues of untreated wild type mice and 4 week-cuprizone feeding wild type and Cys F KO mice, respectively. CXCL2 mRNA and protein were measured by real-time quantitative PCR and ELISA. The results are shown in **(A,B)**, respectively. ^*^P < 0.05, ^**^P < 0.01. *n* = 5 per group.

Thus far, we found the following in Cys F KO mice after cuprizone treatment: (1) aggravated demyelination status; (2) Cat C was mainly derived from microglia/macrophages; and (3) a greater number of leukocytes including monocytes/macrophages accumulated in the demyelinated areas. Cat C has been proved to take part in the release of chemokines, and Cat C gene could deficiency decreases the release of CXCL2 in the peripheral circulation (Pagano et al., 2007). Because CXCL2 is produced by leukocytes (Gu et al., 1999) in the circulatory system, and by microglia/macrophages (Wang et al., 2000; Rouault et al., 2013) and astrocytes (Wang et al., 2014) in the CNS, we were curious about the level of CXCL2 expression in wild and Cys F KO mice after cuprizone treatment. Therefore, real-time quantitative PCR and ELISA were preformed to detect the expression of CXCL2 mRNA and protein, respectively. There were no significant differences between untreated wild type and Cys F KO mice (Supplementary Figures 5A,B), while after 4 weeks of cuprizone feeding, the expression of both CXCL2 mRNA and protein were prominently increased compared to untreated mice, and the highest level of CXCL2 existed in Cys F KO mice (Figures 5A,B). These results suggest that cuprizone treatment significantly increased CXCL2 expression, which might be related to increased accumulation of microglia/macrophages in cuprizone treated Cys F KO mice.

### Cat C stimulated glial cells to produce CXCL2

**Figure 6:**
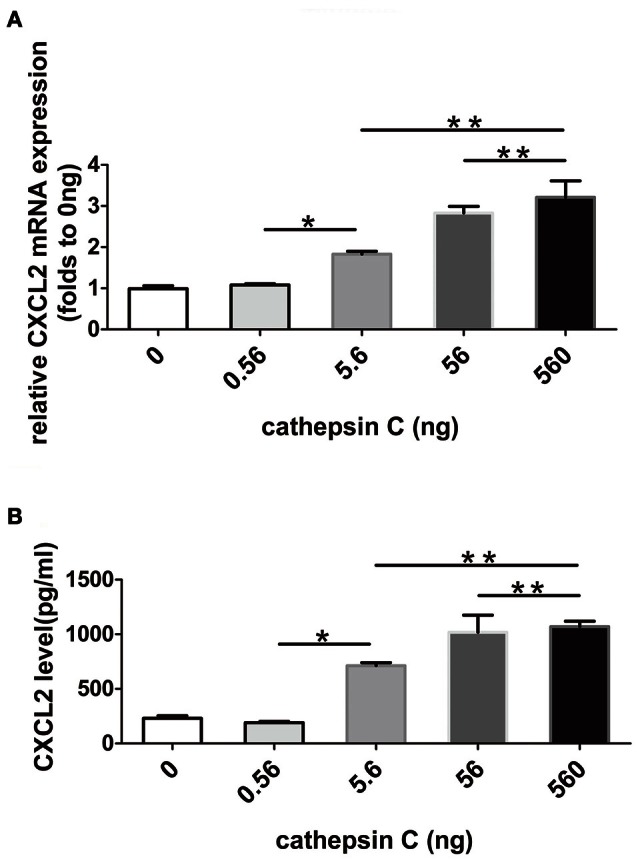
**The expression and release of CXCL2 in mix-cultured glial cells following active recombinant Cat C stimulation**. CXCL2 mRNA and protein expression in mix-cultured glial cells stimulated by active recombinant Cat C in different concentrations were measured by real-time quantitative PCR and ELISA, respectively. The results are shown in **(A,B)**. ^*^P < 0.05, ^**^P < 0.01. *n* = 5 per group.

Because the major inhibitory target of Cys F is Cat C, and up-regulated expression of Cat C has been found in Cys F KO mice, all of these findings implied a possible correlation between Cat C and increased CXCL2 expression. It is known that Cat C is involved in the release of CXCL2 in the peripheral circulation (Pagano et al., 2007). Therefore, we asked whether Cat C was also involved in CXCL2 production in the CNS. We used a recombination monomer of Cat C to stimulate primary cultured glial cells *in vitro*, then we found that both mRNA and protein of CXCL2 was increased with increased doses of Cat C. (Figures 6A,B) These results suggested that Cat C stimulates glial cells to produce CXCL2 *in vitro*.

### Cat C gene knock down decreased CXCL2 expression and accumulation of microglia/macrophages

**Figure 7:**
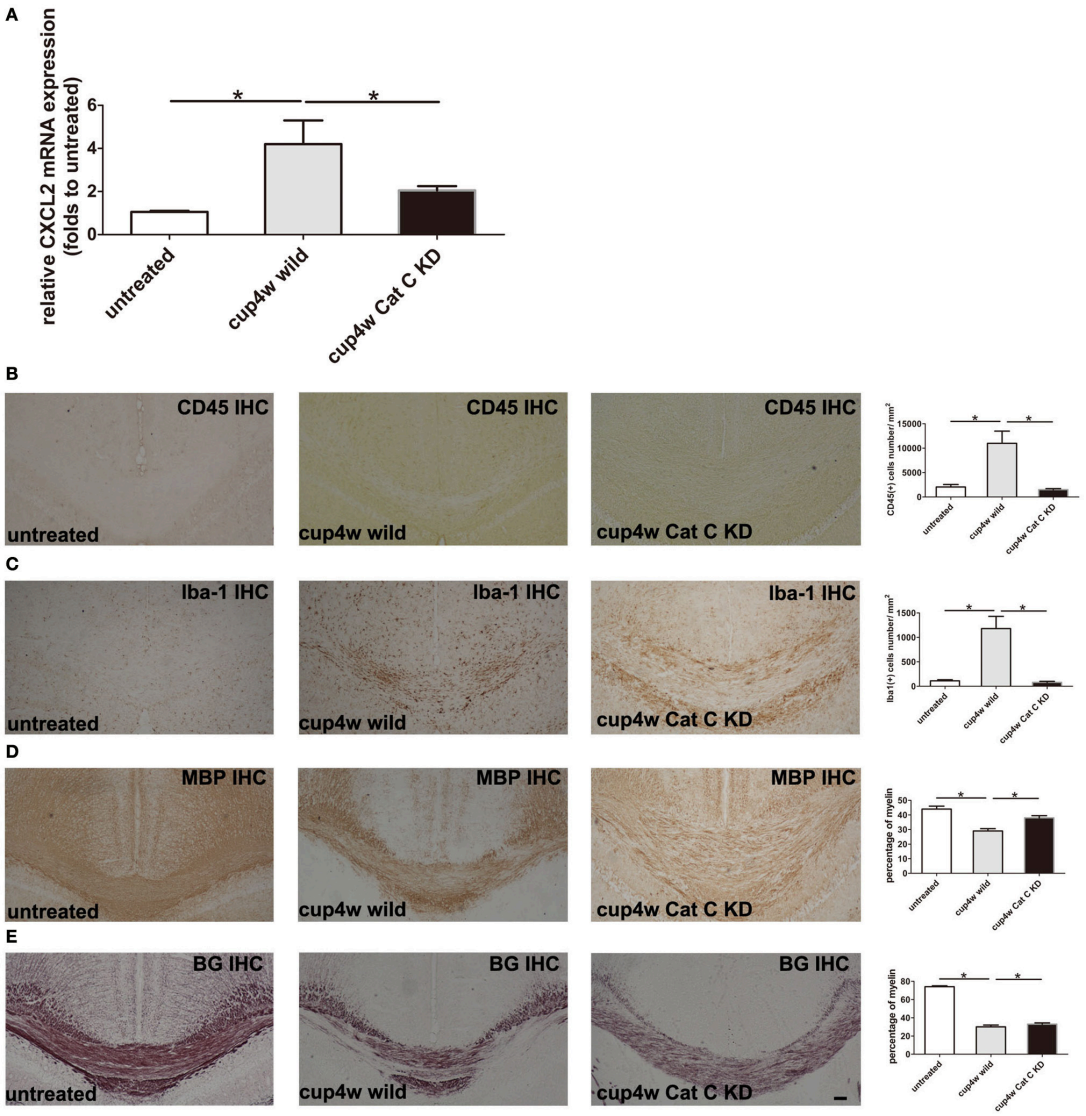
**Demyelination in Cat C KD mice was relieved accompanying decreased CXCL2 expression and the infiltration of bone marrow-derived cells after 4-week cuprizone treatment. (A)** The expression of CXCL2 mRNA were measured by real-time quantitative PCR. **(B)** CD45 IHC positive cells in corpus callosum. **(C)** Iba-1 IHC positive cells in corpus callosum. **(D)** The remaining myelin status in corpus callosum shown by MBP IHC staining. **(E)** The remaining myelin in corpus callosum shown by Black Gold stainning. ^*^P < 0.05, *n* = 3 per group. Scale bar: 100 μm.

Based on the aforementioned results, we raised the hypothesis that a lack of the Cys F gene removed inhibition of Cat C, thus resulting in more chemokine CXCL2 release from glial cells and accumulation of inflammatory cells and led to severe demyelination. To testify our hypothesis, we used transgenic mouse lines in which Cat C gene expression can be manipulated by the FAST system (Tanaka et al., 2010). Because of the STOP sequence forcing termination of transcription of Cat C, the homozygotes of the STOP-tetO knock-in alleles in the absence of the tTA allele (Cat C^*STOP*−*tetO*/*STOP*−*tetO*^) should behave as Cat C knockdown (KD) mice (submitted for publication). We used Cat C KD mice to generate the cuprizone model again. We found no significant differences in CXCL2 mRNA expression between untreated wild type and Cat C KD mice (Supplementary Figure 5C). In addition, there were no apparent differences in myelin status shown by MBP IHC and Black Gold staining (Supplementary Figures 1D–F,G,I,J), but after 4 weeks of cuprizone feeding, CXCL2 mRNA expression was significantly decreased in Cat C KD mice compared to wild type mice (Figure 7A). Additionally, both the CD45-positive and Iba-1-positive cells were also decreased (Figures 7B,C). More importantly, as we expected, the demyelination in Cat C KD mice was relieved (Figures 7D,E).

## Discussion

In the present study, severe demyelination and inflammatory responses were observed in cuprizone-treated Cys F KO mice, implying that Cys F deficiency resulting in disinhibition of Cat C and subsequent up-regulation of CXCL2 expression might aggravate the demyelination in the cuprizone model.

Cys F belongs to type II cystatin, which is selectively expressed in immune cells and is secreted as an inactive disulphide-linked dimer until it is reduced to its monomeric form with proteolytic cleavage on its extended N-terminal region (Cappello et al., 2004; Langerholc et al., 2005). Cys F can strongly inhibit Cat C activity and co-localize with Cat C in murine mast cells and the Cat C substrate granzyme A in human CD8 T cells (Hashimoto et al., 2000). Furthermore, our previous studies showed that in demyelinating animal models, Cys F is co-localized with Cat C, but not co-localized with cathepsin S and L in microglia/macrophages (submitted for publication), although Cys F tightly inhibited cathepsins S and L in other cell types (Hashimoto et al., 2000). Cat C, or dipeptidyl peptidase I (DPPI), modulates inflammatory responses by activating serine proteases in a number of inflammation models (Pham and Ley, 1999; Wolters et al., 2001; Adkison et al., 2002; Pottier et al., 2007; Akk et al., 2008). Specifically, Cat C knockout mice are completely resistant to acute arthritis in a collagen-induced rheumatoid arthritis model (Adkison et al., 2002; Hu and Pham, 2005) and a lack of Cat C alleviates development of experimental abdominal aortic aneurysms by reducing the release of CXC-chemokine ligand (CXCL)2 (Pagano et al., 2007; Shi, 2007). Furthermore, in our previous study, in addition to the demyelination process, up-regulated Cat C was also found in LPS-induced neuroinflammation (Fan et al., 2012). These findings suggest that Cat C might be the key factor causing severe demyelination in cuprizone-treated Cys F KO mice.

The cuprizone model is commonly used in the study of demyelinating diseases due to the reproducible, localized, and predictable features. Cuprizone-induced demyelination is thought to be caused by selective toxicity of cuprizone to oligodendrocytes, specifically through disruption in the mitochondrial complex IV of myelin-forming cells (Acs et al., 2013; Bénardais et al., 2013). In this toxin model, microglia/macrophages actively contribute to the demyelination process (Liu J. et al., 2015). These microglia/macrophages accumulate in the demyelinated areas even though the integrity of the blood-brain barrier is intact in the cuprizone model (McMahon et al., 2001). Furthermore, some studies have demonstrated that circulating CXCR2-positive neutrophils are also important for a two-hit process of cuprizone-induced demyelination (Liu et al., 2010). In the present study, we found a significantly increased number of microglia/macrophages accumulated in demyelinated areas in Cys F KO mice, which may be associated with chemoattraction mediated by CXCL2. CXCL2, a member of the CXC chemokine family, also called macrophage inflammatory protein 2-alpha (MIP2-alpha), is produced not only by leukocytes (Gu et al., 1999) in the circulatory system, but also by microglia/macrophages (Wang et al., 2000; Rouault et al., 2013) and astrocytes (Wang et al., 2014) in the CNS. CXCL2 mobilizes cells by interacting with a cell surface chemokine receptor (CXCR2), whose expression has been identified on many types of resident cells in the CNS, including neurons and glial cells (Goczalik et al., 2008), and leukocytes, including neutrophils, monocytes, and T cells in the peripheral system (Liu Y. et al., 2015).

The previous studies found that CXCL2 expression was significantly induced in encephalomyelitis mouse model with chronic demyelination (Rubio et al., 2006). Moreover, CXCL2 level was up-regulated in rat brain cell cultures subjected to lysophosphatidylcholine (LPC) accompanying obvious demyelination and inflammatory reactivity (Defaux et al., 2010). In our study, CXCL2 expression was induced in mixed-cultured glial cells following treatment of exogenous Cat C *in vitro*. *In vivo*, up-regulated Cat C expression was found in Cys F KO mice after 4 weeks of cuprizone feeding, suggesting that despite of proteolitic properties, Cat C might not be directly involved in myelin sheath protein degradation, but could play a unique role in the demyelination process, such as stimulating glial cells to secrete CXCL2 and thereby attract more inflammatory cells. In addition, these infiltrated cells might express more Cat C protein because we and others have demonstrated that Cat C can be produced by leukocytes in the peripheral system and microglia in the CNS (Fan et al., 2012; Vidal et al., 2013). Therefore, we assumed that significantly up-regulated expression of Cat C after 4-week cuprizone treatment was associated with more recruitment of inflammatory cells expressing excessive Cat C, which was consistent with “two-hit” course (Liu et al., 2010). This assumption can be strengthened by the results showing that CXCL2 level, inflammatory cell number and demyelinating degree were significantly reduced in the cuprizone model of Cat C KD mice, compared with wild type mice.

## Conclusions

Cys F deficiency leading to disinhibition of Cat C aggravates the demyelination in the cuprizone model, which might be associated with up-regulated CXCL2 expression and resulting attraction of inflammatory cells. Cat C and Cys F could be a promising therapeutic target for alleviating demyelination.

## Author contributions

JM and KF conceived of the study, participated in its design and coordination and helped to draft the manuscript. KI participated in the design of the study. JL carried out the immunoassays and drafted the manuscript. NL and XY carried out the RT-PCR. CH and YZ carried out morphological staining and animal treatments. TS and XW performed the statistical analysis. All authors read and approved the final manuscript.

### Conflict of interest statement

The authors declare that the research was conducted in the absence of any commercial or financial relationships that could be construed as a potential conflict of interest.

## Supplementary material

Supplementary Figure 1 **The myelin status in untreated mice including wild type, Cys F KO and Cat C KD mice**. PLP IHC staining was performed in the untreated wild type and Cys F KO mice **(A,B)**; MBP IHC staining was performed in the untreated wild type and Cat C KD mice **(D,E)**; Black Gold staining was performed in the untreated wild type, Cys F KO and Cat C KD mice **(G–I)**. Percentage of remaining myelin in corpus callosum is shown in **(C,F,J)**. *n* = 6 per group. Scale bar in **(A,B,D,E,G–I)**: 200 μm.

Supplementary Figure 2 **The negative expression of Cat C mRNA in corpus callosum in untreated wild type and Cys F KO mice**. Cat C ISH staining was performed in the untreated wild type mice **(A)** and Cys F KO mice **(B)**. *n* = 6 per group. Scale bar in **(A,B)**: 200 μm.

Supplementary Figure 3 **Themicroglia/macrophagesin corpus callosum in untreated wild type and Cys F KO mice**. Iba-1 IHC **(A,B)** and *c-fms* ISH **(D,E)** staining were performed in untreated wild type and Cys F KO mice. Quantitative analyses of Iba-1 and *c-fms* positive cells in corpus callosum are shown in **(C,F)**. *n* = 4 per group. scale bar in **(A,B,D,E)**:200 μm.

Supplementary Figure 4 **CD45 positive expression in untreated wild type and Cys F KO mice**. CD45 IHC staining was performed in untreated wild type mice **(A)** and Cys F KO mice **(B)**, respectively. Quantitative analysis of CD45 positive cells in corpus callosum is shown in **(C)**. *n* = 4 per group. scale bar in **(A,B)**: 200 μm.

Supplementary Figure 5 **The expression of CXCL2 mRNA and protein in the brain of untreated wild type, Cys F KO and Cat C KD mice**. Real time quantitative PCR data are shown in **(A,C)**, ELISA result in **(B)**. *n* = 6 per group.
