# Correction: Disinhibition of Cathepsin C Caused by Cystatin F Deficiency Aggravates the Demyelination in a Cuprizone Model

**DOI:** 10.3389/fnmol.2018.00115

**Published:** 2018-04-12

**Authors:** Junjie Liang, Ning Li, Yanli Zhang, Changyi Hou, Xiaohan Yang, Takahiro Shimizu, Xiaoyu Wang, Kazuhiro Ikenaka, Kai Fan, Jianmei Ma

**Affiliations:** ^1^Graduate School of Dalian Medical University, Dalian, China; ^2^Cardiovascular Division, Hailar People's Hospital, Hailar, China; ^3^Department of Surgery, Wafangdian Central Hospital, Dalian, China; ^4^Department of Anatomy, Dalian Medical University, Dalian, China; ^5^Liaoning Provincial Key Laboratory of Brain Diseases, Dalian, China; ^6^Richardson Lab, University College London, London, United Kingdom; ^7^Department of Linguistics and Modern Languages, Chinese University of Hong Kong, Shatin, Hong Kong, China; ^8^Department of Neurobiology and Bioinformatics, National Institute for Physiological Sciences, Aichi, Japan

**Keywords:** cystatin F, cathepsin C, microglia, CXCl2, demyelination, cuprizone

In the original article as published, there was a mistake for grant number (**81271322**) of National Natural Science Foundation of China in acknowledgments. The correct grant number should be **81671180**. The grant number for Natural Science Foundation of Liaoning Province (2015020256) is no problem, and need not correction. The authors apologize for the mistake. This error does not change the scientific conclusions of the article in any way.

The original article has been updated.

## Conflict of interest statement

The authors declare that the research was conducted in the absence of any commercial or financial relationships that could be construed as a potential conflict of interest.

