# Supplementary figures and images for: Disinhibition of Cathepsin C Caused by Cystatin F Deficiency Aggravates the Demyelination in a Cuprizone Model
Source: Front Mol Neurosci. 2016 Dec 21;9:152. doi: 10.3389/fnmol.2016.00152 (PMC5175397; doi:10.3389/fnmol.2016.00152)

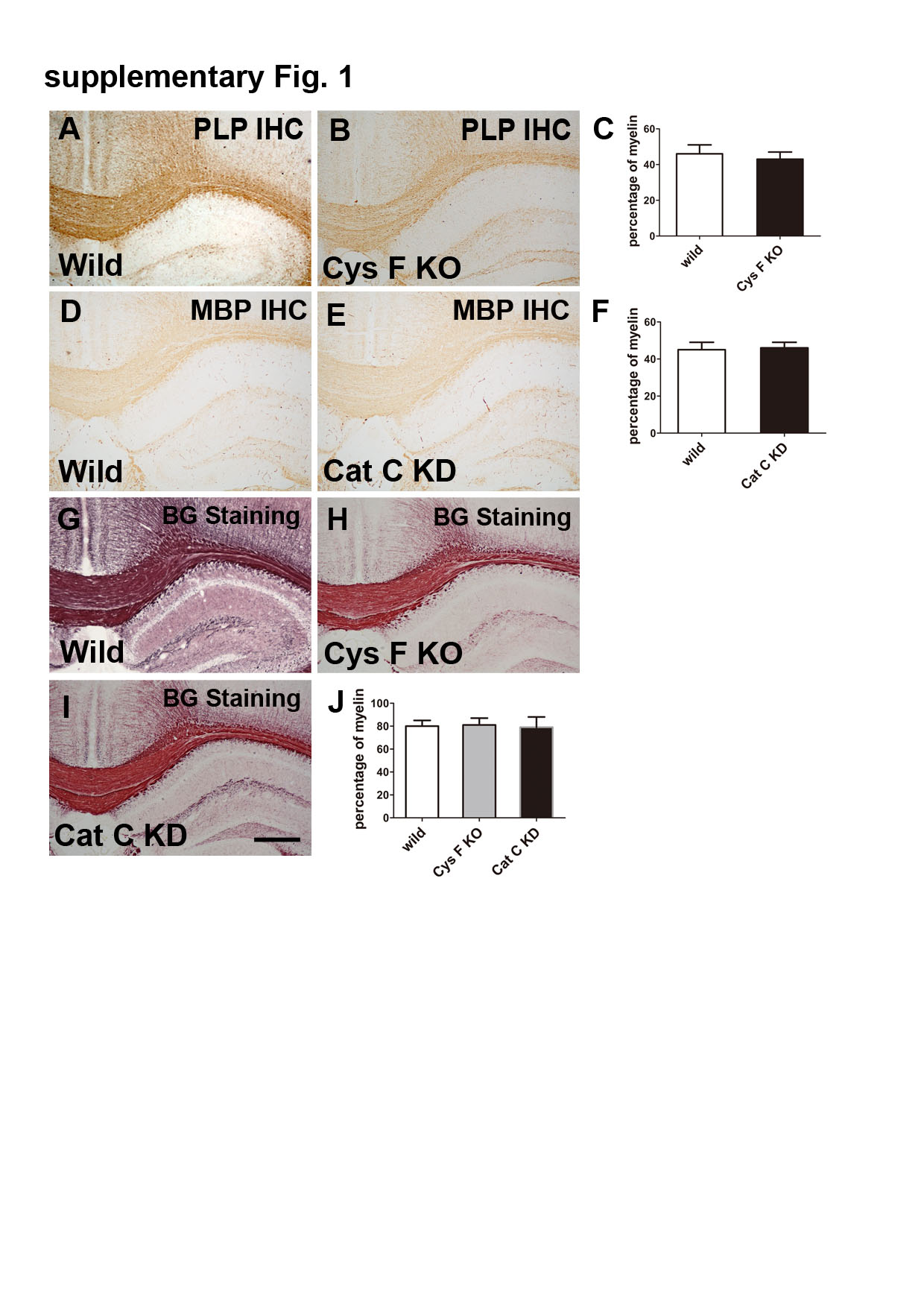

Supplement: Supplementary Figure 1 — The myelin status in untreated mice including wild type, Cys F KO and Cat C KD mice. PLP IHC staining was performed in the untreated wild type and Cys F KO mice (A,B); MBP IHC staining was performed in the untreated wild type and Cat C KD mice (D,E); Black Gold staining was performed in the untreated wild type, Cys F KO and Cat C KD mice (G–I). Percentage of remaining myelin in corpus callosum is shown in (C,F,J). n = 6 per group. Scale bar in (A,B,D,E,G–I): 200 μm. [file Image1.JPEG]

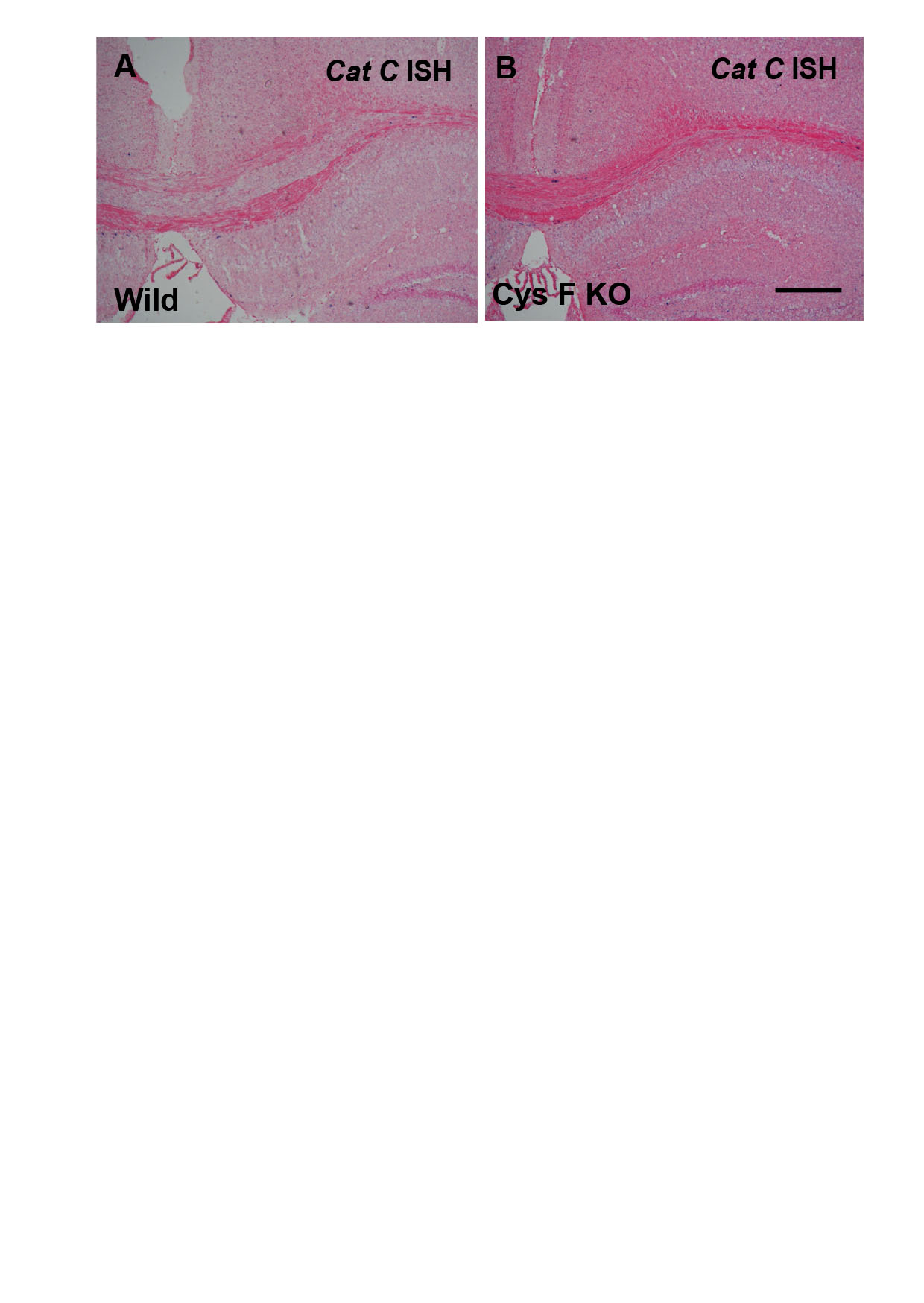

Supplement: Supplementary Figure 2 — The negative expression of Cat C mRNA in corpus callosum in untreated wild type and Cys F KO mice. Cat C ISH staining was performed in the untreated wild type mice (A) and Cys F KO mice (B). n = 6 per group. Scale bar in (A,B): 200 μm. [file Image2.JPEG]

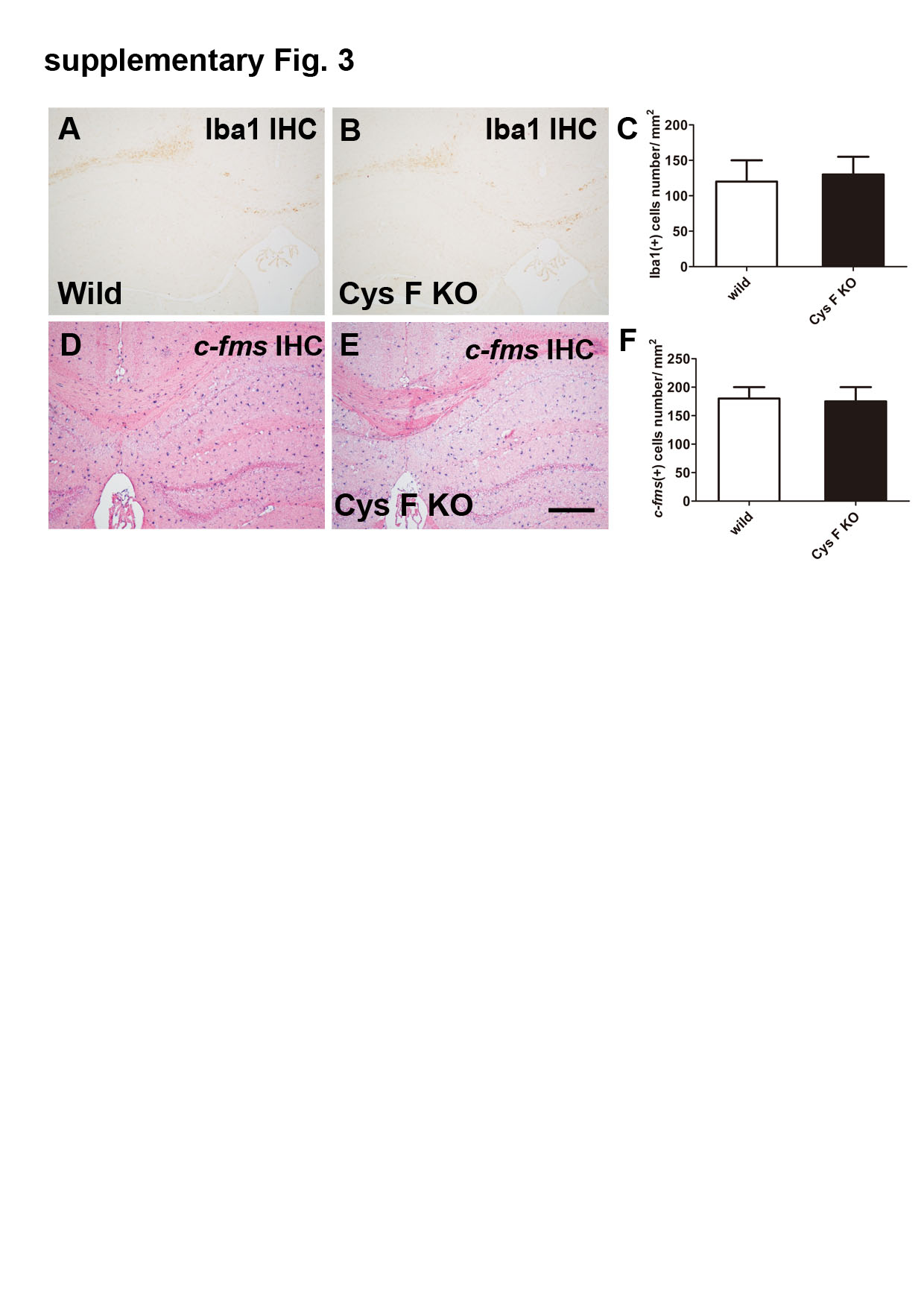

Supplement: Supplementary Figure 3 — Themicroglia/macrophagesin corpus callosum in untreated wild type and Cys F KO mice. Iba-1 IHC (A,B) and c-fms ISH (D,E) staining were performed in untreated wild type and Cys F KO mice. Quantitative analyses of Iba-1 and c-fms positive cells in corpus callosum are shown in (C,F). n = 4 per group. scale bar in (A,B,D,E):200 μm. [file Image3.JPEG]

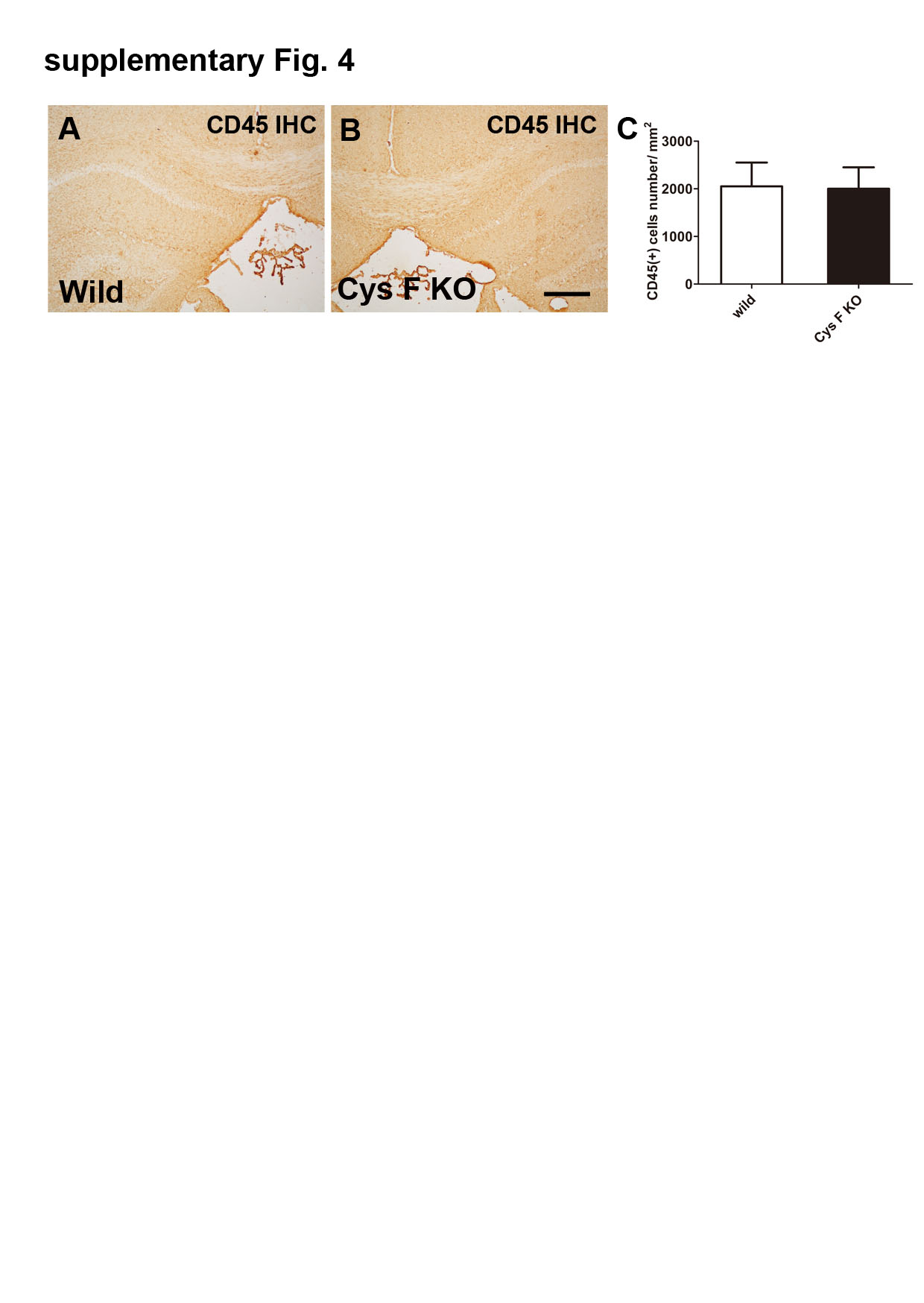

Supplement: Supplementary Figure 4 — CD45 positive expression in untreated wild type and Cys F KO mice. CD45 IHC staining was performed in untreated wild type mice (A) and Cys F KO mice (B), respectively. Quantitative analysis of CD45 positive cells in corpus callosum is shown in (C). n = 4 per group. scale bar in (A,B): 200 μm. [file Image4.JPEG]

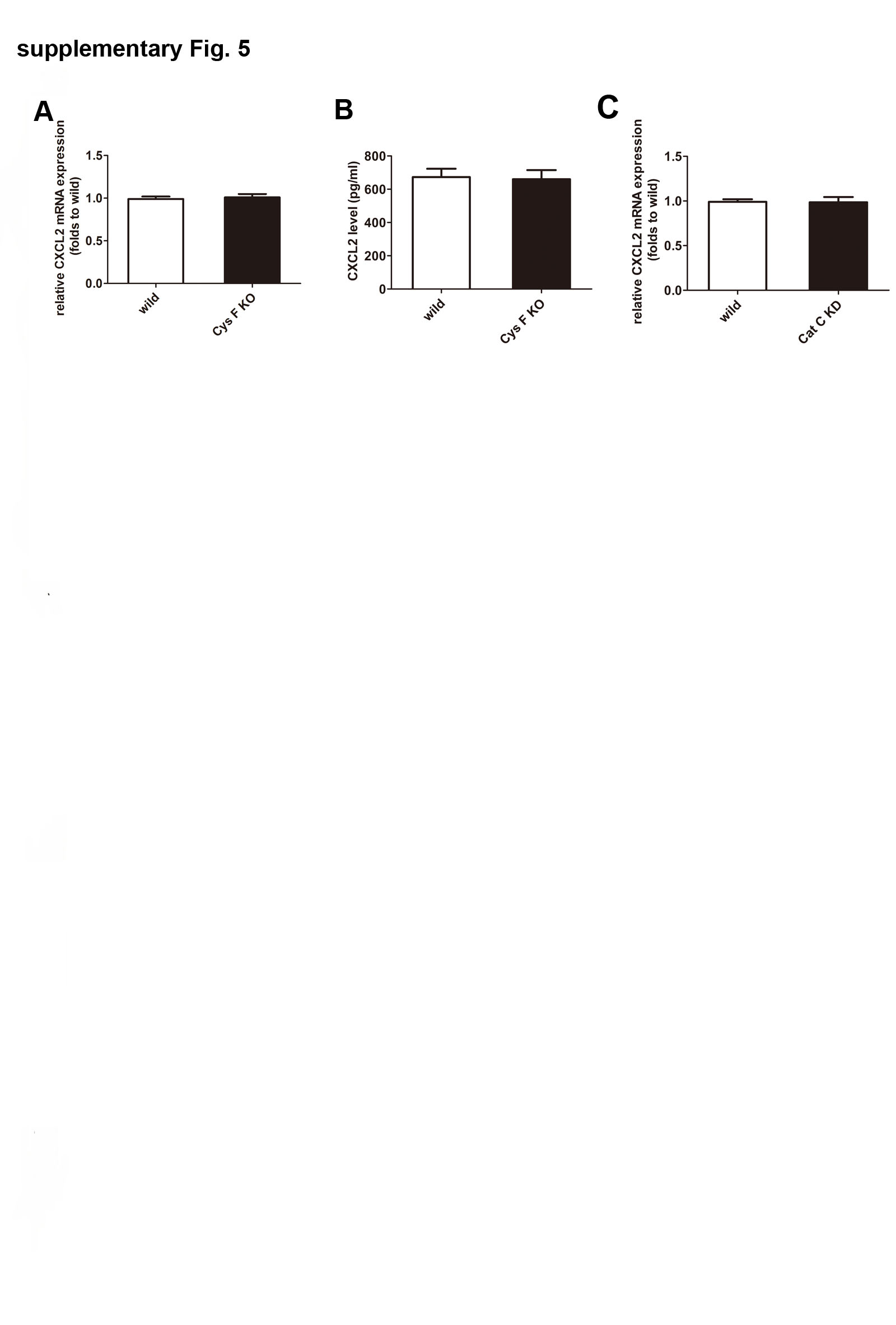

Supplement: Supplementary Figure 5 — The expression of CXCL2 mRNA and protein in the brain of untreated wild type, Cys F KO and Cat C KD mice. Real time quantitative PCR data are shown in (A,C), ELISA result in (B). n = 6 per group. [file Image5.JPEG]
